# Association Between Helicobacter Pylori Infection and Long-term Outcome in Patients with Drug-eluting Stent Implantation

**DOI:** 10.1038/srep44954

**Published:** 2017-07-13

**Authors:** Rui Wang, Lei-lei Chen, De-zhao Wang, Bu-xing Chen

**Affiliations:** 1 Department of Cardiology, Beijing Tiantan Hospital, Capital Medical University, Beijing 100050, China

## Abstract

To investigate the relationship between Helicobacter pylori (Hp) infection and the long-term outcome in acute coronary syndrome (ACS) patients with drug-eluting stent (DES) implantation and so as to explore the significance of Hp eradication therapy in preventing major adverse cardiac events (MACE) and upper gastrointestinal bleeding (UGIB). 539 ACS patients with DES implantation from January 1, 2010 to December 31, 2012 were analyzed. All the patients were divided into two groups according to the result of ^13^C urea breath test. 253 patients with Hp infection were put into group A (Hp^+^), and 286 cases without Hp infection were put into group B (Hp^−^). Demographic data was collected and all patients went through biochemical indicators and other routine blood examinations. We explored the correlations of Hp infection with MACE and UGIB after 3 to 5 years of follow-up using survival analysis. Survival analysis showed that Hp infection was a predictor of MACE and UGI. Sub-group analysis showed that patients with Hp eradication therapy had no relationship with MACE but had a lower rate of UGIB than those without Hp eradication therapy.

Helicobacter pylori (Hp) infection has been suspected as a pathogenic factor in many diseases. It is a frequent cause of dyspepsia, esophageal motility disorders^1^, gastro-esophageal reflux disease (GERD), peptic ulcer disease (PUD) and upper gastrointestinal bleeding (UGIB)^2^,^3^. As a precipitating factor of atherosclerotic plaque progression and instability, Hp infection may play a potential role in the pathogenesis of chest pain in patients with coronary artery disease (CAD)^4^. Although there are still some doubts over the role of Hp infection in atherosclerosis, some epidemiological studies have shown that Hp seropositivity was significantly and positively associated with the occurrence of acute coronary syndrome (ACS) and atherosclerotic progression^5^. With the widespread use of drug-eluting stents (DES) in ACS patients, studies have shown that the incidence rate of UGIB was increasing year by year in the patients who receive dual anti-platelet treatment. The rates for patients taking and not taking dual antiplatelet drugs to develop UGIB are respectively 12.5% and 4.0%^6^,^7^. Many ACS patients receiving dual anti-platelet therapy also suffer from Hp infection, which have greatly increased the incidence of UGIB. Major adverse cardiac events (MACE) is the primary end point, which includes all-cause mortality, myocardial infarction and target vessel revascularization. Previous studies have demonstrated that triple antiplatelet treatment after stent implantation was associated with feasible benefits on reducing the risk of MACE^8^.

The Maastricht IV/Florence Consensus Report shows that those who taking dual anti-platelet drugs with Hp infection do not need the Hp eradication therapy if with no symptoms in digestive system^9^. However, it is still controversial over whether these asymptomatic patients with Hp infection should take eradication therapy or not, and this issue is not mentioned in the guideline. Radical treatment requires increased medication types and patient compliance. Whether Hp eradication therapy for patients with DES could reduce the UGIB and major adverse cardiac events (MACE) or not is still unclear.

The purpose of this study was to investigate the relationship between Hp infection and the long-term outcome of ACS patients who take dual anti-platelet therapy for one year after DES implantation, and the effects of Hp eradication therapy on the incidence of MACE and UGIB through a long-term follow-up.

## Materials and Methods

### Subjects

#### Study design and setting

The following methods were carried out in accordance with the approved guidelines. All authors reviewed the results and approved the final version of the manuscript. This study was approved by the Ethics Committee of the Beijing Tiantan Hospital.

All experimental protocols were approved by Beijing Tiantan Hospital, and the written informed consent was obtaining from every patient. This study was a prospective, two-center trial. 572 ACS patients, who received DES implantation with dual antiplatelet therapy, hospitalized in Beijing Tiantan Hospital and Mentougou District Hospital from January 1, 2010 to December 31, 2012 were selected. Expect 33 patients lost to follow-up, a total of 539 patients with complete clinical data were collected in the study, and all the enrolled patients matched the inclusion and exclusion criteria. The disease history, physical examination, and laboratory results of patients were recorded. 539 subjects, with 184 males (34.1%) and 355 females (65.9%) were divided into two groups according to the result of ^13^C urea breath test. 253 patients with Hp infection (positive of ^13^C urea breath test) were set into group A (Hp^+^), and 286 cases without Hp infection were set into group B (Hp^−^). Group A (Hp^+^) was further divided into two sub-groups (with/without Hp eradication therapy) using the digital random method.

#### Inclusion criteria

All the subjects met the following criteria: (1) with DES implantation following dual anti-platelet therapy; (2) having experienced ^13^C urea breath test during hospitalization; and (3) having not received Hp eradication therapy before.

#### Exclusion criteria

Subjects who had suffered gastrointestinal bleeding, or with a history of gastrectomy, cardiac insufficiency, thyroid dysfunction, and any ongoing infections were eliminated. Patients who had used antibiotics, bismuth, proton pump inhibitor or sucralfate within one month were also excluded. Those who had gastrointestinal symptoms such as acid reflux, heartburn, nausea, vomiting, stomach ache and diarrhea, or had a confirmed peptic ulcer with Hp infection were also considered ineligible because they might have received gastroenterology treatment.

### Tests and examinations

#### Routine examination

All patients underwent the following tests/examinations the next day after admission: blood routine examination, glycosylated hemoglobin, hs-CRP, HCY, blood coagulation, ^13^C urea breath, and ultrasonic cardiogram (UCG). Subjects were asked to keep 8 hours of fasting for blood test.

#### ^13^C urea breath test

The ^13^C urea breath test was conducted in the morning using the HCBT-01 breath test automatic instrument (Shenzhen Zhonghe Haidewei Biological Technology Co. Ltd) and Hp infection was determined by ^13^C/^12^C isotope ratio (δ‰). Subjects were asked to achieve 2 hours of fasting, and specialized personnels were in strict accordance with the operating requirements. The positive value of Hp infection was defined as δ ≥ 4 ± 0.4‰.

### Coronary angiography (CAG) and percutaneous coronary intervention (PCI)

All patients underwent diagnostic CAG, which was performed based on the standard institution protocol, with 5 views of the left coronary, 2 views of the right coronary, and 2 orthogonal views of the target lesion. De-identified angiographic data sets were analyzed by a single interpreter. Minimal lumen diameter (MLD) was measured in the view with the greatest degree of stenosis. Proximal and distal reference diameters were measured and averaged to calculate the percentage of the diameter stenosis (%DS).

All patients with acute ST-segment elevation myocardial infarction (STEMI) underwent therapeutic reperfusion using primary PCI. For all patients with non-ST-segment elevation myocardial infarction (NSTEMI) and unstable angina pectoris (UAP) the elective PCI was performed. Loading doses of asprin (300 mg) and clopidogrel (300 mg) were administered immediately after signing informed consent before taking PCI. Anticoagulation was achieved with unfractionated heparin (100 IU/kg), and an activated clotting time ≥250 seconds was maintained. Aspiration thrombectomy, balloon angioplasty and stenting implantation were performed using standard techniques. Predilatation, direct stenting and post-stenting balloon inflation were performed at the operator’s discretion. The procedure was considered successful if the residual stenosis was <25% with a grade of 3 in Thrombolysis in Myocardial Infarction (TIMI) flow.

### Eradication therapy

Eradication therapy was composed of rabeprazole (10 mg, twice daily), colloidal bismuth subcitrate (240 mg, twice daily), amoxicillin (1000 mg, twice daily), and clarithromycin (500 mg, twice daily) for ten days. If patients were allergic to penicillin, then amoxicillin was replaced with metronidazole (400 mg twice daily). ^13^C urea breath test should be performed at least for 4 weeks after the treatment ended, and patients should stop taking rabeprazole 2 weeks before the review for that false negative might be caused. If the initiative therapy failed, another regimen that was composed of rabeprazole (10 mg, twice daily), colloidal bismuth subcitrate (240 mg, twice daily), furazolidone (100 mg, twice daily) and tetracycline (500 mg, 4 times daily) for ten days would be selected as the rescue therapy.

### Follow-up visits

All patients were followed up for 3 to 5 years at an outpatient clinic after hospital discharge. Follow-up visits with a cardiologist were scheduled 1 month after discharge and then every 6 month till the end of the study. During the follow-up, patients were monitored for MACE (including myocardial infarction, target vessel revascularization, and all-cause death) and UGIB, which were considered as end-points. Myocardial infarction (MI) was defined as chest pain with new ST-segment changes and elevation of cardiac markers which reflected myocardial necrosis to at least twice the upper limit of normal. PCI-related MI was not included as clinical events in this study. Target vessel revascularization (TVR) was defined as clinically driven percutaneous revascularization or bypass of the target lesion or any segment of the epicardial coronary artery that contained the target lesion. Otherwise, measurements were obtained at the end of the study. After completing data collection in all patients, we studied the association between the variables collected on admission and patient outcome after 3–5years.

### Ethics

The study was approved by the Ethics Committee of Beijing Tiantan Hospital and Mentougou District Hospital. Informed consent was obtained for all the subjects and signed by themselves.

### Statistical methods

Baseline characteristics of patients were compared using t-test for continuous variables and the chi-squared test for non-continuous variables. When continuous data was not normally distributed, groups were compared using nonparametric Wilcoxon rank sum test. The correlation between baseline characteristics and ACS was tested using a cox proportional hazards regression model. Clinically significant variables were adjusted and entered into a multivariate regression model using the method of backward stepwise: wald. *P* < 0.05 was deemed statistically significant. Statistical analysis was performed using the SPSS software package (version 19.0).

## Results

### The baseline data of the two groups

There was no significant difference in the baseline data between group A (Hp^+^) and B (Hp^−^) (Table 1). There were 126 patients receiving Hp eradication therapy. ^13^C urea breath test was performed at least for 4 weeks after the treatment ended to estimate the therapeutic effects. The results of UBT test demonstrated that the initiative therapy was effective for the 126 patients, and none of them needed the rescue therapy.

### Comparison of MACE and UGIB between the two groups

**Figure 1:**
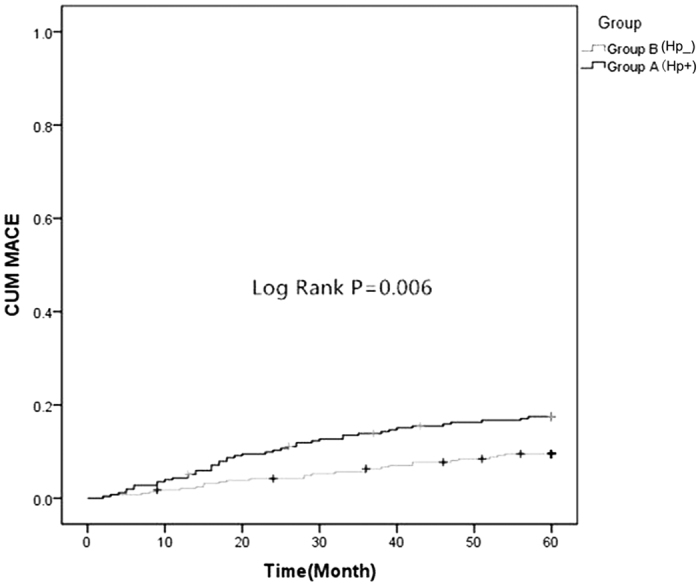
Comparison of two Kaplan-Meier curves as a function of time to the MACE between group A (Hp^+^) and group B (Hp^−^) (Log Rank test, F = 7.699, *P* = 0.006).

**Figure 2:**
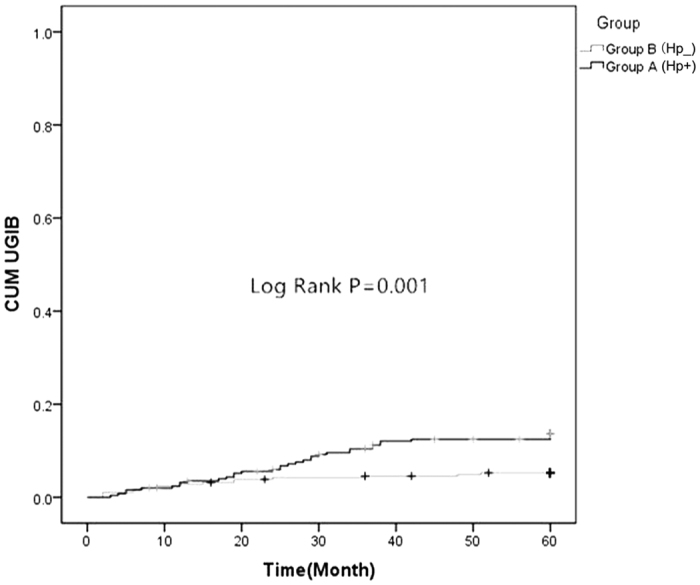
Comparison of two Kaplan-Meier curves as a function of time to the UGIB between group A (Hp^+^) and group B (Hp^−^) (Log Rank test, F = 10.852, *P* = 0.001).

**Figure 3:**
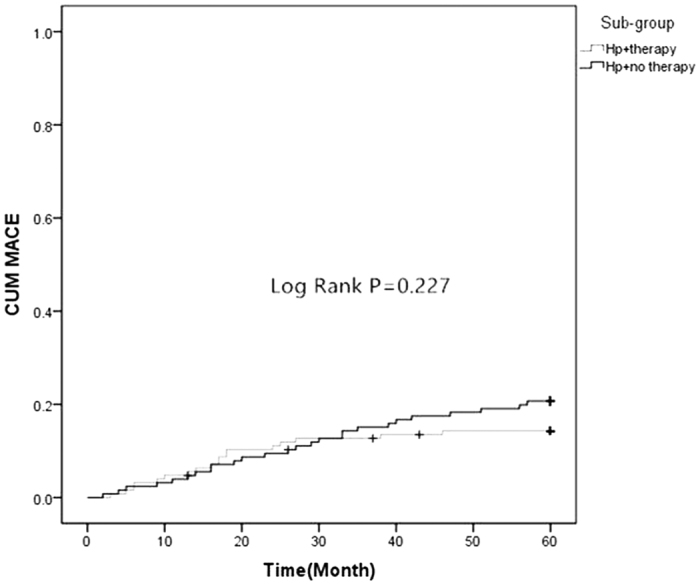
Comparison of two Kaplan-Meier curves as a function of time to the MACE between Hp-positive patients with eradication therapy (Hp + Therapy) and Hp-positive patients without eradication therapy (Hp + No Therapy) (Log Rank test, F = 1.460, *P* = 0.227).

**Figure 4:**
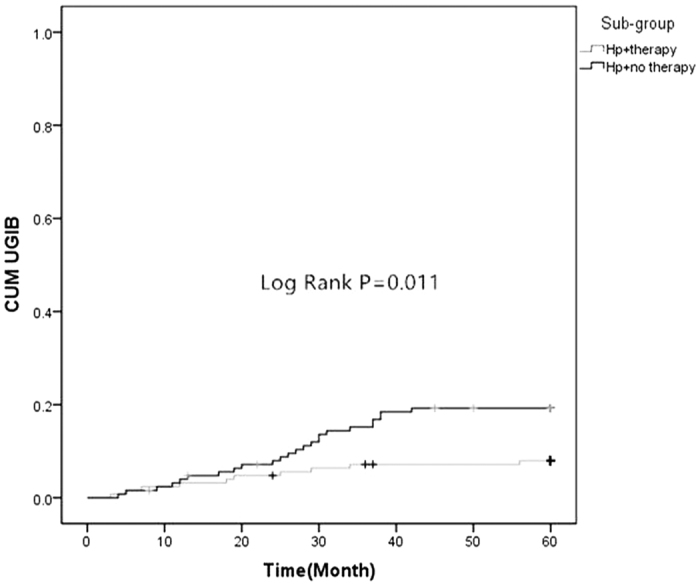
Comparison of two Kaplan-Meier curves as a function of time to the UGIB between Hp-positive patients with eradication therapy (Hp + Therapy) and Hp-positive patients without eradication therapy (Hp + No Therapy) (Log Rank test, F = 6.469, *P* = 0.011).

There were no significant differences in the prevalence of MI and revascularization between group A (Hp^+^) and B (Hp^−^). But Group A (Hp^+^) had more MACE and UGIB than group B (Hp^−^) (17.4% *vs.* 9.1%, *P* = 0.004; 13.4% *vs.* 5.2%, *P* = 0.001) (Table 2, Figs 1 and 2). There were no significant differences in the prevalence of death, MI and revascularization in sub-groups (Table 3 and Fig. 3). However, Hp + no therapy group had more UGIB than Hp + therapy group (18.1% *vs.* 8.7%, *P* = 0.029) (Table 3 and Fig. 4).

### Muti-factorial analysis using the Cox proportional hazard model in patients with MACE and UGIB

In the multivariate Cox regression model, after adjusting for other important covariates, Hp infection remained an independent predictor for both MACE and UGIB (harzard ratio (HR) = 2.010, 95% confidence interval (95% CI) = 1.109–3.645; HR = 2.609; 95% CI = 1.419–4.795 (Table 4). In the sub-groups, Hp infection + no therapy was an independent predictor for MACE and UGIB (HR = 3.437, 95% CI = 1.461–8.809; HR = 2.607, 95% CI: 1.246–5.455) (Table 5).

## Discussion

In this study, we first evaluated the effects of Hp infection and conventional cardiovascular disease risk factors on the incidence of future MACE among 539 patients presenting with ACS. The results reflected a strong association between Hp infection and the incidence of MACE in participants during the 3 to 5 years of follow-up after hospital discharge. However, we did not find any significant association between Hp infection and other studied risk factors. The statistically significant effect on the MACE in ACS patients was confirmed in survival analysis (Fig. 1) and multi-factorial analyses (Table 4). Sub-group analysis showed that patients with Hp eradication therapy had no relationship with MACE compared with those without Hp eradication therapy.

Our findings were consistent with majority of the previous studies, which also involved mixed subject groups, such as patients with normal coronary angiogram or significant narrowing of the coronary arteries. Significant correlation of Hp infection with ACS and cardiac syndrome X has ever been demonstrated in previous studies^7^,^8^,^9^,^10^,^11^,^12^,^13^,^14^,^15^,^16^,^17^, but contrary results have also been reported in other researches^18^,^19^,^20^. Hp is a pathogen that can cause persistent infection even be life-long, and antibodies could be persistently generated. Several pathological mechanisms have been postulated to explain the effect of Hp infection in atherosclerosis. It has been suggested that Hp initiates an acute-phase response and activates TNF-α, IL-6 and fibrinogen inflammatory cytokines, which could directly or indirectly propagate an inflammatory process in the arterial walls^21^,^22^. In addition, it can cause platelet aggregation, an important aspect of acute destabilization of atherosclerotic disease^23^. Hp may also lead to endothelial damage though aggravated autoimmune hormonal responses caused by antigenic mimicry^24^ as well as the immunological cross reactivity between bacterial and human heat-shock proteins^25^. This process may lead to coronary calcification and atherosclerosis^26^. In addition, researcher have found Hp in the arterial vascular wall^19^, and in the atherosclerotic plaque using polymerase chain reaction (PCR) technique^27^. Despite some conflicting data has been reported^28^, we considered that the discrepancy may be due to differences in the study populations and detection method^29^. In our study, we used ^13^C urea breath test to detect Hp infection.

The study of Eskandarian *et al*.^29^ showed that Hp IgA seropositivity was significantly associated with fatal cardiovascular events, with a hazard ratio of 2.58, which is similar to the findings in our study. As mentioned above, Hp has been shown to cause platelet aggregation^23^. During the acute phase of ACS and plaque disruption, this could lead to local inflammation and aggravated platelet aggregation, which is a crucial cause to acute myocardial ischemia. This process may explain why Hp infection was associated with long-term outcomes. Additionally, our results showed that no significant difference on MACE incidence was found between the Hp-positive patients with eradication therapy and Hp-positive patients without eradication therapy (14.3% *vs.* 20.5%, *P* = 0.194; Table 3 and Fig. 3). Therefore, it was concluded that Hp eradication therapy could not reduce the incidence of MACE. Moreover, this study also demonstrated a positive association between Hp infection and the incidence of UGIB in the participants during follow-up. It was statistically significant, as confirmed in the survival analysis (Fig. 2) and the multi-factorial analyses (Table 4). The Hp positive subjects without eradication therapy were at a 2.607-fold greater risk for UGIB than subjects with eradication therapy. Subjects who were Hp-positive with eradication therapy had significantly different outcome compared to the subjects who were Hp-positive without eradication therapy (18.1% *vs.* 8.7%, *P* = 0.029) (Table 3 and Fig. 4).

In our study, no significant association was found between the conventional cardiac risk factors (hypertension, DM, hyperlipidemia, smoking, and positive family history) and the incidence of MACE. This was consistent with the results reported by Eskandarian *et al*., who did not demonstrate a prognostic role of these classic risk factors on MACE in patients with ACS either^29^. These results may be partly due to aggressive treatment of these risk factors in patients diagnosed with ACS in the study population. Longer follow-up duration and a larger number of sample populations may be needed in future to further evaluate the prognostic effects of these factors.

Earlier studies have shown that history of peptic ulcer, cardiogenic shock, cardiac arrest, inotropic agent requirement, and primary PCI are important risk factors for UGIB occurrence in patients with CAD^4^,^30^. However, the Hp infection was not shown to be an important risk factor for UGIB in patients with ACS in these studies^4^,^31^. Hp infection is an important risk factor for peptic ulcer bleeding in those who receive aspirin therapy^32^. Our study has shown that Hp infection was an independent risk factor for UGIB in ACS patients after DES implantation with dual antiplatelet therapy, and Hp eradication therapy could reduce the incidence of UGIB. The divergence between the results of our study and previous studies may due to differences in the selected populations, study design, and variations in the follow-up. The molecular mechanism about the Hp eradication therapy reducing the incidence of UGIB in ACS patients with DES will be studied in the future.

## Conclusion

The results obtained in this study have shown that the ACS patients with Hp infection had more coronary events and UGIB in the first 3 to 5 years after DES implantation. Hp eradication therapy could reduce the incidence of UGIB.

## Limitation

The limitation of our study included relatively smaller sample size and less rigorous inclusion criteria. In addition, not all patients except those with UGIB required endoscopic follow-up.

## Additional Information

**How to cite this article:** Wang, R. *et al*. Association Between Helicobacter Pylori Infection and Long-term Outcome in Patients with Drug-eluting Stent Implantation. *Sci. Rep.* **7**, 44954; doi: 10.1038/srep44954 (2017).

**Publisher's note:** Springer Nature remains neutral with regard to jurisdictional claims in published maps and institutional affiliations.

## Figures and Tables

**Table 1:** Baseline data of the two groups.

	Group A (Hp^+^) n = 253	Group B (Hp^−^) n = 286	*P* value
Females, n (%)	160 (63.2)	195 (68.4)	0.227
Smoke, n (%)	129 (49.0)	143 (50.0)	0.819
Hypertension, n (%)	135 (53.4)	147 (51.4)	0.649
Dyslipedemia, n (%)	104 (41.1)	103 (36.0)	0.225
Diabetes Melitus, n (%)	74 (29.2)	74 (25.9)	0.381
Cerebrovascular disease, n (%)	21 (8.3)	31 (10.8)	0.319
Family history of CAD, n (%)	16 (6.3)	13 (4.5)	0.361
Fundus abnormal, n (%)	225 (88.9)	249 (87.1)	0.799
Coronary artery lesions, n (%)			0.882
Single vessel lesion	105 (41.5)	124 (43.4)	
Double or left main lesion	71 (28.1)	80 (28.0)	
Triple vessel lesion	77 (30.4)	82 (28.7)	
ACS, n (%)			0.425
UAP	162 (64.0)	198 (69.2)	
AMI	91 (36.0)	88 (30.8)	
Primary PCI, n (%)	87 (34.4)	94 (32.9)	0.709
Asprin, n (%)	249 (98.4)	283 (99.0)	0.870
Clopidogrel, n (%)	251 (99.2)	281 (98.3)	0.549
Mean age ± SD (years)	61.3 ± 11.32	60.6 ± 10.80	0.411
Mean stent ± SD (mm)	35.7 ± 14.45	37.9 ± 15.10	0.166
Mean SBP ± SD (mmHg)	134.7 ± 21.93	137.8 ± 23.85	0.114
Mean HR ± SD (beats/min)	74.2 ± 11.72	74.9 ± 12.98	0.458
Mean WBC ± SD (10^9^/L)	6.9 ± 2.71	7.1 ± 2.65	0.537
Mean LVEF ± SD (%)	60.9 ± 7.50	61.1 ± 7.41	0.874
Mean BMI ± SD (kg/m^2^)	24.7 ± 3.52	25.1 ± 3.51	0.094
Median hs-CRP (mg/L) (interquartile range)	2.90 (1.58–8.45)	2.06 (1.20–5.77)	0.202
Median HCY (umol/L) (interquartile range)	12.00 (8.56–18.60)	11.2 (6.66–17.20)	0.075

Abbreviations: CAD, coronary artery disease; ACS, acute coronary syndrome; UAP, unstable angina pectoris; AMI, acute myocardial infarction; PCI, percutaneous coronary intervention; SD, standard deviation; SBP, systolic blood pressure; HR, heart rate; WBC, white blood-cell count; LVEF, left ventricular ejection fraction; BMI, body mass index; hs-CRP, high sensitive C-reactive protein; HCY, homocysteine.

**Table 2:** Clinical events from PCI until end of follow up by two groups.

	Group A (Hp^+^) n = 253	Group B (Hp^−^) n = 286	*P* value
Follow-up (cumulated events), n (%)			
Death	26 (10.3)	12 (4.2)	0.006
MI	17 (6.7)	10 (3.5)	0.087
Revascularization	12 (4.7)	6 (2.1)	0.088
MACE (death/MI/revascularization)	44 (17.4)	26 (9.1)	0.004
UGIB, n (%)	34 (13.4)	15 (5.2)	0.001

Abbreviations: MI. myocardial infarction; MACE, major adverse cardiac events; UGIB, upper gastrointestinal bleeding.

**Table 3:** Clinical events from PCI until end of follow up by sub-groups.

	Hp + Therapy n = 126	Hp + No Therapy n = 127	*P* value
Follow-up (cumulated events), n (%)			
Death	11 (8.7)	14 (11.0)	0.541
MI	9 (7.1)	8 (6.3)	0.257
Revascularization	4 (3.2)	9 (7.1)	0.159
MACE (death/MI/revascularization)	18 (14.3)	26 (20.5)	0.194
UGIB, n (%)	11 (8.7)	23 (18.1)	0.029

Abbreviations: MI. myocardial infarction; MACE, major adverse cardiac events; UGIB, upper gastrointestinal bleeding; Hp+Therapy, Hp positive patients with Hp eradication therapy; Hp+No Therapy, Hp positive patients without Hp eradication therapy.

**Table 4:** Cox regression analysis in patients with MACE and UGIB (Method: Backwrd Stepwise-Wald).

Category	B value	SE	Wald X^2^	*P* value	HR (95%CI)
MACE					
Hp infection	0.698	0.304	5.287	0.021	2.010 (1.109–3.645)
UGIB					
HCY (umol/L)	0.023	0.012	3.490	0.062	1.023 (0.999–1.047)
Hp infection	0.959	0.311	9.534	0.002	2.609 (1.419–4.795)

Abbreviations: MACE, major adverse cardiac events; UGIB, upper gastrointestinal bleeding; Hp, Helicobacter pylori; HCY, homocysteine; SE, standard error; HR, hazard ratio; CI, confidence interval.

**Table 5:** Cox regression analysis in the sub-groups patients with MACE and UGIB (Method: Backwrd Stepwise-Wald).

Category	B value	SE	Wald X^2^	*P* value	HR (95%CI)
MACE					
Hp infection+no therapy	1.235	0.437	7.994	0.005	3.437 (1.461–8.809)
LVEF	0.079	0.028	7.719	0.005	1.082 (1.023–1.143)
UGIB					
Hp infection+no therapy	0.958	0.377	6.467	0.011	2.607 (1.246–5.455)

Abbreviations: MACE, major adverse cardiac events; UGIB, upper gastrointestinal bleeding;LVEF, left ventricular ejection fraction; Hp, Helicobacter pylori; SE, standard error; HR, hazard ratio; CI, confidence interval.
